# Self-Neglect: One Pathway to Surrogate Decision-Making

**DOI:** 10.1093/geroni/igaa057.2468

**Published:** 2020-12-16

**Authors:** Holly Ramsey-Klawsnik, Jason Burnett

**Affiliations:** 1 Self-Employed, Canton, Massachusetts, United States; 2 McGovern Medical School at UTHealth, Houston, Texas, United States

## Abstract

Self-neglect, the most frequently reported allegation to Adult Protective Services (APS), involves profoundly harmful behaviors often due to functional or cognitive limitations, health problems, and insufficient resources that result in older adults insufficiently meeting their basic needs. Outcomes include high risk of illness, hospitalization and readmission, hospice and nursing home use, early mortality, and placement under surrogate decision-making authority of either well-intended or opportunistic others. APS staff are charged with assessing self-neglect and intervening to reduce client danger. A nationwide APS survey revealed program policies, procedures, resources, and needs affecting the client welfare. For example, 92% of APS programs have provisions for seeking guardianship for self-neglecting individuals, in 25% of programs staff serve as court-appointed guardians, and a wide variety of tools are used within APS programs to assess clients’ mental capacity. Key study findings, implications, and recommendations will be presented.

